# Supplementary material for: Duloxetine and cognitive behavioral therapy with phone-based support for the treatment of chronic musculoskeletal pain: study protocol of the PRECICE randomized control trial
Source: Trials. 2024 May 18;25:330. doi: 10.1186/s13063-024-08158-x (PMC11102257; doi:10.1186/s13063-024-08158-x)
Supplement: Supplementary file 2 — Additional file 2: Ethical approval document [file 13063_2024_8158_MOESM2_ESM.doc]

# MEMORANDUM

______________________________________________________________________________________

To: Dennis Ang, M.D., M.S.

Int Med-Rheumatology

From: Richard Weinberg, M.D., Chair, IRB # 4

Institutional Review Board

Date: 5/28/2020

Subject: Human Protocol: IRB00065428

Pain Response Evaluation of a Combined Intervention to Cope Effectively

Study Documents:

Protocol Version: PRECICE.protocol 5_13_2020.docx; Informed Consent Version: IC.PRECICE5.21.20.docx; Investigator's Brochure: Package insert Duloxtine.pdf; Other Documents: Study intervention.docx, Study questionnaires.docx

The Institutional Review Board (IRB) has approved the above-named protocol and study documents, after review at a convened meeting on 5/21/2020. A submission requesting renewal together with a summary progress report must be submitted to the Board at least one month prior to 5/20/2021.

This submission has met the requirements of the 2019 Common Rule.

This approval includes a limited waiver of HIPAA authorization to identify potential subjects for recruitment into this research study, as allowed under 45 CFR 164.512. This temporary waiver provides access to protected health information (PHI) to confirm eligibility and facilitate initial contact, after which consent and HIPAA autorization will be sought. Access and use is limited to the minimum amount of PHI necessary to review eligibility criteria and to contact potential subjects.

A waiver for the requirements of signed consent and HIPAA authorization have been granted by the IRB for preliminary screening purposes.

Federal regulations and Board policy require that you promptly report to the Board for review/approval:

· Proposed changes in the research activity (e.g., protocol amendments; consent form revision; advertisements). Changes may not be initiated without IRB review and approval, unless necessary to eliminate an immediate hazard to subjects.

· Serious adverse events and unanticipated problems involving risks must be reported to the Board, institutional officials, FDA, sponsor and other regulatory agencies as required by the protocol, local policy and state or federal regulation.

Please provide a final report to the Board when the project is completed and Board approval can be terminated.

The Wake Forest School of Medicine IRB is duly constituted, has written procedures for initial and continuing review of clinical trials; prepares written minutes of convened meetings, and retains records pertaining to the review and approval process; all in compliance with requirements of FDA regulations 21 CFR Parts 50 and 56, HHS regulations 45 CFR 46, and International Conference on Harmonisation (ICH) E6, Good Clinical Practice (GCP), as applicable. WFSM IRB is registered with OHRP/FDA; our IRB registration numbers are IRB00000212, IRB00002432, IRB00002433, IRB00002434, IRB00008492, IRB00008493, IRB00008494, and IRB00008495.

WFSM IRB has been continually fully accredited by the Association for the Accreditation of Human Research Protection Programs (AAHRPP) since 2011.

[
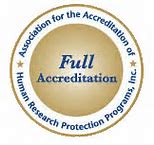
](https://www.bing.com/images/search?view=detailV2&ccid=V2Udk7Tg&id=ED1D0E7880D532C1CD7055BD910D208BBD55A79B&thid=OIP.V2Udk7TgkLcyrbJzZnCaUgAAAA&mediaurl=https%3A%2F%2Fwww.guthrie.org%2Fsites%2Fdefault%2Ffiles%2FAAHRPP-Logo.gif&exph=153&expw=162&q=aahrpp+logo&simid=608044102151309059&selectedIndex=0)
