# Supplementary material for: Duloxetine and cognitive behavioral therapy with phone-based support for the treatment of chronic musculoskeletal pain: study protocol of the PRECICE randomized control trial
Source: Trials. 2024 May 18;25:330. doi: 10.1186/s13063-024-08158-x (PMC11102257; doi:10.1186/s13063-024-08158-x)
Supplement: Supplementary file 3 — Additional file 3: Model consent form [file 13063_2024_8158_MOESM3_ESM.pdf]

Wake Forest®
School of Medicine

Department/Section of Internal Medicine/Rheumatology

Pain Response Evaluation of a Combined Intervention to Cope Effectively

Informed Consent Form to Participate in Research
Dennis Ang, M.D. Principal Investigator

SUMMARY

You are invited to participate in a research study. The purpose of this research is is to determine if the combination of non-opioid medication (duloxetine) and web-based pain-coping skills training (PCST) is beneficial for individuals with chronic musculoskeletal pain (CMP). You are invited to be in this study because you have chronic musculoskeletal pain in different body parts. Your participation in this research will involve 4 visits and last about 2 hours.

Participation in this study will involve completing the symptom questionnaires, web modules, and daily intake of an FDA-approved non-addicting medication (duloxetine) for chronic musculoskeletal pain. All research studies involve some risks. A risk to this study that you should be aware is that you may (or may not) experience self-limiting duloxetine side effects such as nausea and sweating; and feeling uncomfortable talking to us over the phone. There is the possibility that you may benefit from participation in this study.

Your participation in this study is voluntary. You do not have to participate in this study if you do not want to. There may be other choices available to you. Some other choices may include follow-up with your primary care doctor for pain management. You will not lose any services, benefits, or rights you would normally have if you choose not to participate.

The remainder of this form contains a more complete description of this study. Please read this description carefully. You can ask any questions if you need help deciding whether to join the study. The person in charge of this study is Dennis Ang, M.D. If you have questions, suggestions, or concerns regarding this study or you want to withdraw from the study his/her contact information is: 336-716-0186.

If you have any questions, suggestions or concerns about your rights as a volunteer in this research, contact the Institutional Review Board at 336-716-4542 or the Research Subject Advocate at Wake Forest at 336-716-8372.

WFU School of Medicine
Institutional Review Board
IRB Number:IRB00065428
Meeting Date Approved 5/21/2020
Version Valid Until: 5/20/2021

INTRODUCTION

You are invited to be in a research study. Research studies are designed to gain scientific knowledge that may help other people in the future. You are being asked to take part in this study because you have chronic musculoskeletal pain in different body parts. Please take your time in making your decision as to whether or not you wish to participate. Ask your study doctor or the study staff to explain any words or information contained in this informed consent document that you do not understand. You may also discuss the study with your friends and family.

WHY IS THIS STUDY BEING DONE?

The primary purpose of the study is to determine if the combination of non-opioid medication (duloxetine) and web-based pain-coping skills training (PCST) is beneficial for individuals with chronic musculoskeletal pain (CMP). The secondary purpose is to determine if a nurse-assisted phone delivered coaching is more effective than no coaching in reducing pain and improving physical function therapy.

While you take part in this study, you may not take part in any other medical research studies, however observational studies can be allowed. If you want to take part in another study, please inform your study doctor.

HOW MANY PEOPLE WILL TAKE PART IN THE STUDY?

280 people at Wake Baptist Health System will take part in this study.

WHAT IS INVOLVED IN THE STUDY?

Your study doctor will continue to make treatment decisions. This study will not influence how your study doctor chooses to treat your condition.

Study Visit(s)
If you agree to participate, before any information is collected, you will be asked to review and sign this informed consent document.

After you have been informed about the study and signed this form, your study doctor will collect information concerning your health, medical diagnoses and medications you are taking at each visit.

Baseline Visit:

You will come to the research clinic to provide information on your medical health and all the medications you are taking. You will also complete baseline questionnaires; and have your height, weight, urine pregnancy test (if relevant), and blood pressure checked. Thereafter, you will be randomly assigned to either one of these 3 treatment arms:

(1) Duloxetine and web based pain-coping skills training (PCST) with nurse support,
(2) Duloxetine and web based pain-coping skills training (PCST) without nurse support,
(3) Duloxetine only

WFU School of Medicine
Institutional Review Board
IRB Number:IRB00065428
Meeting Date Approved 5/21/2020
Version Valid Until: 5/20/2021

Neither the study doctor nor the research recruitment personnel will know which of the treatments you are receiving; however, this information can be made available if medically necessary.

(1) Duloxetine and web based pain-coping skills training with nurse support

If you are randomized to this treatment arm, you will receive a medication packet of duloxetine (30 mg per capsule) with the instruction to take one capsule once daily for one week. After one week, you will return to the research clinic to receive a medication packet with 90 capsules of duloxetine 60 mg with the instruction to take one capsule once daily for 12 weeks. You will also be completing web-based pain-coping skills, an automated program that includes eight 35- to 45-minute training sessions, which will help you better manage your pain. You will go through the 8 learning modules over a 12-week period. In addition, you will receive six phone calls from our research nurse to motivate you to apply the newly learned pain coping skills on your daily life.

(2) Duloxetine and web-based pain-coping skills training without nurse support

If you are randomized to this treatment arm, you will receive a medication packet of duloxetine 30 mg once daily for one week. After one week, you will return to the research clinic to receive a medication packet with 90 capsules of duloxetine 60 mg with the instruction to take one capsule once daily for 12 weeks. You will also be completing web-based pain-coping skills, an automated program that includes eight 35- to 45-minute training sessions, which will help you better manage your pain. You will go through the 8 learning modules over a 12-week period. You will not receive a phone call from our research nurse.

(3) Duloxetine only

If you are randomized to this treatment arm, you will receive a medication packet of duloxetine 30 mg once daily for one week. After one week, you will return to the research clinic to receive a medication packet with 90 capsules of duloxetine 60 mg with the instruction to take one capsule once daily for 12 weeks. You do not need to complete the web-based pain-coping skills training. You will not receive a phone call from our research nurse.

To assure your safety, a research staff will call you to complete a medication side-effect checklist. You will receive the phone call weekly for 2 weeks, and every 4 weeks for 2 months. Every adverse event will be reported to the study Medical Safety Officer.

Week 13

You will return to the research clinic to complete symptom-based questionnaires including: brief pain inventory/pain severity and physical function scale, pain catastrophizing scale, global rating of change, and anxiety and depression scales. You will have your weight, and blood pressure checked. You will be asked to get a urine pregnancy test, if relevant. You will also be asked to review any new or existing adverse events and update your medications. All participants will continue duloxetine (60 mg per

WFU School of Medicine
Institutional Review Board
IRB Number:IRB00065428
Meeting Date Approved 5/21/2020
Version Valid Until: 5/20/2021

capsule) one capsule once daily for the next 12 weeks.

If you were previously assigned to the treatment group with access to the web-based pain coping skills training, you will maintain access to the website over the next 12 weeks. Access to this website will allow you to review and practice those newly learned pain coping skills.

Week 25 (final outcome assessment)

You will come to the research clinic and complete another set of symptom-based questionnaires. You will have your weight, and blood pressure checked.

- If you want to continue with duloxetine, you will be given a 1-week supply of duloxetine 60 mg. The 1-week supply will give you enough time to ask your primary care provider (PCP) to request for a refill. In addition, the project manager will send a note to your PCP with a short summary about the research study including a statement that you tolerated duloxetine very well.
- If you do not want to continue with duloxetine, you will be provided with duloxetine (30 mg per capsule) one capsule once daily for one week, and then discontinue the medication.

The study does not require any specific testing. This study does not change the way in which your doctor delivers your care.

As part of this research study, you will be audiotaped. This is being done for training and quality assurance purposes. You understand that you may request the recording be stopped at any time during the course of the research study. You can also withdraw your consent to use and disclose the audiotapes before it is used. You should also understand that you will not be able to inspect, review, or approve the audiotapes or other media (including articles containing such) before they are used in this study.

Please choose one of the following regarding the use and disclosure of the audiotapes used in this research study:

______ I would like the audiotapes of me to be destroyed once their use in this study is finished.

_____ The audiotapes of me can be kept for use in future studies provided they are kept secure and any future study will be reviewed by an IRB. I understand that I will not be able to inspect, review or approve their future use.

HOW LONG WILL I BE IN THE STUDY?

You will be in the study for about 24 weeks. You can stop participating at any time. If you decide to stop participating in the study we encourage you to talk to the investigators or study staff first to learn about any potential health or safety concerns. There are no serious consequences if you should suddenly withdrawal from the study.

WFU School of Medicine
Institutional Review Board
IRB Number:IRB00065428
Meeting Date Approved 5/21/2020
Version Valid Until: 5/20/2021

Wake Forest®
School of Medicine

WHAT ARE THE RISKS OF THE STUDY?

The risks primarily involve direct adverse effects of duloxetine which are well-described and minimal (i.e., sedation, nausea, headache, and dizziness that are typically transient in nature) in most individuals. For those who do not tolerate the side effects, the medication will be discontinued with an appropriate 7-day tapering regimen to prevent discontinuation syndrome. Side effects of these medications resolve shortly after stopping them.

Reproductive Risks and other Issues to Participating in Research

Pregnant women are excluded from participation in this study. Because NO method of birth control is 100% reliable, a pregnancy test is required at baseline and at your third study visit (week 13).

If you happen to get pregnant while in the study, the **rare** side effects of duloxetine for the fetus late in the third trimester include breathing difficulties, seizures, temperature instability, feeding difficulty, vomiting, low blood sugar, jitteriness, irritability, and tremor. To avoid unnecessary exposure to duloxetine, you will get a repeat urine pregnancy test mid-way (week 13) during the 24-study period.

Other potential risks include emotional distress related to the web-based pain coping skills training, which is typically a minor issue. Our trained research assistant, who collects information on medication side effects, will be able to ask about emotional distress, with an agreed upon approach to refer you to the medical safety officer if any concerns are present. You should discuss the risk of being in this study with the study staff.

There are no additional tests performed while enrolled in this study; however, your study doctor may capture results of testing performed for your normal clinical care.

Taking part in this research study may involve providing information that you consider confidential or private. Efforts, such as coding research records, keeping research records secure and allowing only authorized people to have access to research records, will be made to keep your information safe.

ARE THERE BENEFITS TO TAKING PART IN THE STUDY?

You may experience relief of pain and improvement in quality of life. It is possible; the information learned from this study will benefit other people in the future.

WHAT OTHER CHOICES ARE THERE?

Your alternative is to not participate in this study.

WFU School of Medicine
Institutional Review Board
IRB Number:IRB00065428
Meeting Date Approved 5/21/2020
Version Valid Until: 5/20/2021

What Are the Costs?

All study costs related directly to the study will be paid for by the study. Costs for your regular medical care, which are not related to this study, will be your own responsibility.

Will Your Research Records be Confidential?

The results of this research study may be presented at scientific or medical meetings or published in scientific journals. Your identity and/or your personal health information will not be disclosed unless it is authorized by you, required by law, or necessary to protect the safety of yourself or others. There is always some risk that even de-identified information might be re-identified.

The information collected by the study doctor or study staff as part of the study may be sent to other members to contractors and consultants working for the Sponsor and to regulatory authorities.

Participant information may be provided to Federal and other regulatory agencies as required. The Food and Drug Administration (FDA), for example, may inspect research records and learn your identity if this study falls within its jurisdiction.

If you choose to participate in this study, your medical record at Wake Forest University Baptist Medical Center will indicate that you are enrolled in a research study. Information about the research may be included in your medical record. This part of the medical record will only be available to people who have authorized access to your medical record. If you are not a patient at this Medical Center, a medical record will be created for you anyway to ensure that this important information is available to doctors in case of an emergency.

Will You Be Paid for Participating?

You will be paid $25 for each completed scheduled study visits. If you complete all study visits you will be paid a total of $100. If you withdraw for any reason from the study before completion, you will receive $25 for each study visit you have completed. Cost for your regular medical care, which are not related to this study, will be your own responsibility

To receive payment, you must provide your social security number, name and address so that we can comply with IRS (Internal Revenue Service) reporting requirements. When payments are reported to the IRS we do not let them know what the payment is for, only that you have been paid. If you do not wish to provide this information you can still take part in this study, but you will not be paid.

Who is Sponsoring this Study?

This study is being sponsored by the National Institute of Health. The sponsor is providing money or other support to Wake Forest University Health Sciences to help

WFU School of Medicine
Institutional Review Board
IRB Number:IRB00065428
Meeting Date Approved 5/21/2020
Version Valid Until: 5/20/2021

conduct this study. The researchers do not, however, hold a direct financial interest in the sponsor or the product being studied.

What Happens if You Experience an Injury or Illness as a Result of Participating in this Study?

Should you experience a physical injury or illness as a direct result of your participation in this study, Wake Forest University School of Medicine maintains limited research insurance coverage for the usual and customary medical fees for reasonable and necessary treatment of such injuries or illnesses. To the extent research insurance coverage is available under this policy the reasonable costs of these necessary medical services will be paid, up to a maximum of $25,000. Wake Forest University Baptist Medical Center holds the insurance policy for this coverage. It provides a maximum of $25,000 coverage for each claim and is limited to a total of $250,000 for all claims in any one year. The Wake Forest University School of Medicine, and the North Carolina Baptist Hospitals, Incorporated do not assume responsibility to pay for these medical services or to provide any other compensation for such injury or illness. Additional information may be obtained from the Medical Center's Director of Risk and Insurance Management, at (336) 716-3467.

If you are injured, the insurer may require information such as your name, social security number, and date of birth in order to pay for your care. This is because the insurer is required by law to report any payments made to cover the care of any persons who are members of a government insurance plan to the Department of Health and Human Services.

You do not give up any legal rights as a research participant by signing this consent form. For more information on medical treatment for research related injuries or to report a study related illness, adverse event, or injury you should call Dr. Dennis Ang at (336) 716-0186 during normal business hours and (336) 716-7654.

What about My Health Information?

In this research study, any new information we collect from you and/or information we get from your medical records or other facilities about your health or behaviors is considered <u>Protected Health Information</u>. The information we will collect for this research study includes: your health information, disease, and medications you are taking.

We will make every effort to keep your Protected Health Information private. We will store records of your Protected Health Information in a cabinet in a locked office or on a password protected computer.

Your personal health information and information that identifies you ("your health information") may be given to others during and after the study. Monitors, auditors, IRB or other regulatory agencies will be granted direct access to the participant's original medical record for verification of clinical trial procedures or data, without violating

WFU School of Medicine
Institutional Review Board
IRB Number:IRB00065428
Meeting Date Approved 5/21/2020
Version Valid Until: 5/20/2021

confidentiality of the participant and to the extent permitted by other applicable laws. This is for reasons such as to carry out the study, to determine the results of the study, to make sure the study is being done correctly, to provide required reports and to get approval for new products.

Some of the people, agencies and businesses that may receive and use your health information are the research sponsor; representatives of the sponsor assisting with the research; investigators at other sites who are assisting with the research; central laboratories, reading centers or analysis centers; the Institutional Review Board; representatives of Wake Forest University Health Sciences and North Carolina Baptist Hospital; representatives from government agencies such as the Food and Drug Administration (FDA), the Department of Health and Human Services (DHHS) and similar agencies in other countries.

Some of these people, agencies and businesses may further disclose your health information. If disclosed by them, your health information may no longer be covered by federal or state privacy regulations. Your health information may be disclosed if required by law. Your health information may be used to create information that does not directly identify you. This information may be used by other researchers. You will not be directly identified in any publication or presentation that may result from this study unless there are photographs or recorded media which are identifiable.

If required by law or court order, we might also have to share your Protected Health Information with a judge, law enforcement officer, government agencies, or others. If your Protected Health Information is shared with any of these groups it may no longer be protected by federal or state privacy rules.

Any Protected Health Information collected from you in this study that is maintained in the research records will be kept for at least 15 years. This authorization does not expire and/or any research information entered into your medical record will be kept for as long as your medical record is kept by the Medical Center. You will not be able to obtain a copy of your Protected Health Information in the research records until all activities in the study are completely finished.

You can tell Dennis Ang, M.D. that you want to take away your permission to use and share your Protected Health Information at any time by sending a letter to this address:

Dennis Ang
Wake Forest University Baptist Medical Center
Department of Rheumatology
Medical Center Blvd
Winston Salem, NC, 27157

However, if you take away permission to use your Protected Health Information you will not be able to be in the study any longer. We will stop collecting any more information about you, but any information we have already collected can still be used for the purposes of the research study.

WFU School of Medicine
Institutional Review Board
IRB Number:IRB00065428
Meeting Date Approved 5/21/2020
Version Valid Until: 5/20/2021

By signing this form you give us permission to use your Protected Health Information for this study.

If you choose to participate in this study, your medical record at Wake Forest University Baptist Medical Center will indicate that you are enrolled in a clinical trial. Information about the research and any medications or devices you are being given as a participant may also be included in your medical record. This part of the medical record will only be available to people who have authorized access to your medical record. If you are not a patient at this Medical Center, a medical record will be created for you anyway to ensure that this important information is available to doctors in case of an emergency. A description of this clinical trial will be available on http://www.ClinicalTrials.gov, as required by U.S. Law. This website will not include information that can identify you. At most, the website will include a summary of the results. You can search this Web site at any time.

What Are My Rights as a Research Study Participant?

Taking part in this study is voluntary. You may choose not to take part or you may leave the study at any time. Refusing to participate or leaving the study will not result in any penalty or loss of benefits to which you are entitled. If you decide to stop participating in the study we encourage you to talk to the investigators or study staff first to learn about any potential health or safety consequences. The investigators also have the right to stop your participation in the study at any time. This could be because the study is canceled. Your primary therapy will be stopped if your study doctor believes it is in your best interest to stop treatment for safety or tolerability reasons, such as a side effect.

You will be given any new information we become aware of that would affect your willingness to continue to participate in the study.

Whom Do I Call if I Have Questions or Problems?

For questions about the study or in the event of a research-related injury, contact the study investigator, Dennis Ang at 336-713-4265.

The Institutional Review Board (IRB) is a group of people who review the research to protect your rights. If you have a question about your rights as a research participant, or you would like to discuss problems or concerns, have questions or want to offer input, or you want to obtain additional information, you should contact the Chairman of the IRB at (336) 716-4542.

I have been informed that my study doctor will inform my other doctors, if any, about my participation in this study, and I agree to this.

Yes ☐ No ☐ NA, I have no other doctors ☐

(Please check yes, no, or NA)

You will be given a copy of this signed consent form.

WFU School of Medicine
Institutional Review Board
IRB Number:IRB00065428
Meeting Date Approved 5/21/2020
Version Valid Until: 5/20/2021

SIGNATURES

I agree to take part in this study. I authorize the use and disclosure of my health information as described in this consent and authorization form. If I have not already received a copy of the Privacy Notice, I may request one or one will be made available to me. I have had a chance to ask questions about being in this study and have those questions answered. By signing this consent and authorization form, I am not releasing or agreeing to release the investigator, the sponsor, the institution or its agents from liability for negligence.

Subject Name (Printed):_________________________________

Subject Signature: __________________________________Date: ______Time:_____ am pm

Person Obtaining Consent (Printed):_________________________________

Person Obtaining Consent: _____________________________Date:______Time:_____ am pm

WFU School of Medicine
Institutional Review Board
IRB Number:IRB00065428
Meeting Date Approved 5/21/2020
Version Valid Until: 5/20/2021
