# Supplementary material for: Duloxetine and cognitive behavioral therapy with phone-based support for the treatment of chronic musculoskeletal pain: study protocol of the PRECICE randomized control trial
Source: Trials. 2024 May 18;25:330. doi: 10.1186/s13063-024-08158-x (PMC11102257; doi:10.1186/s13063-024-08158-x)
Supplement: Supplementary file 4 — Additional file 4: Funding document [file 13063_2024_8158_MOESM4_ESM.pdf]

Ph 1 Explor./Developmental Coop. Agreement **Federal Award Date:** 08/14/2020
Department of Health and Human Services
National Institutes of Health

NATIONAL INSTITUTE OF NURSING RESEARCH

Grant Number: 1UG3NR019196-01
FAIN: UG3NR019196

Principal Investigator(s):
Dennis Chua Ang, MD

Project Title: Pain Response Evaluation of a Combined Intervention to Cope Effectively (PRECICE)

Horton, Angela E
OSP Administrator IV
Wake Forest University Health Sciences
Medical Center Boulevard
Winston-Salem, NC 271570001

Award e-mailed to: awards@wakehealth.edu

Period Of Performance:
Budget Period: 09/01/2020 – 08/31/2024
Project Period: 09/01/2020 – 08/31/2024

Dear Business Official:

The National Institutes of Health hereby awards a grant in the amount of $3,929,419 (see “Award Calculation” in Section I and “Terms and Conditions” in Section III) to WAKE FOREST UNIVERSITY HEALTH SCIENCES in support of the above referenced project. This award is pursuant to the authority of 42 USC 241 42 CFR PART 52 and is subject to the requirements of this statute and regulation and of other referenced, incorporated or attached terms and conditions.

Acceptance of this award including the “Terms and Conditions” is acknowledged by the grantee when funds are drawn down or otherwise obtained from the grant payment system.

Each publication, press release, or other document about research supported by an NIH award must include an acknowledgment of NIH award support and a disclaimer such as “Research reported in this publication was supported by the National Institute Of Nursing Research of the National Institutes of Health under Award Number UG3NR019196. The content is solely the responsibility of the authors and does not necessarily represent the official views of the National Institutes of Health.” Prior to issuing a press release concerning the outcome of this research, please notify the NIH awarding IC in advance to allow for coordination.

Award recipients must promote objectivity in research by establishing standards that provide a reasonable expectation that the design, conduct and reporting of research funded under NIH awards will be free from bias resulting from an Investigator’s Financial Conflict of Interest (FCOI), in accordance with the 2011 revised regulation at 42 CFR Part 50 Subpart F. The Institution shall submit all FCOI reports to the NIH through the eRA Commons FCOI Module. The regulation does not apply to Phase I Small Business Innovative Research (SBIR) and Small Business Technology Transfer (STTR) awards. Consult the NIH website http://grants.nih.gov/grants/policy/coi/ for a link to the regulation and additional important information.

If you have any questions about this award, please contact the individual(s) referenced in Section IV.

Sincerely yours,

Brian Albertini
Grants Management Officer
NATIONAL INSTITUTE OF NURSING RESEARCH

Additional information follows

SECTION I – AWARD DATA – 1UG3NR019196-01

**Award Calculation (U.S. Dollars)**	
**Salaries and Wages**	$1,812,327
**Fringe Benefits**	$329,170
**Personnel Costs (Subtotal)**	$2,141,497
**Consultant Services**	$15,300
**Materials & Supplies**	$10,085
**Travel**	$10,000
**Other**	$84,850
**Subawards/Consortium/Contractual Costs**	$396,235

**Federal Direct Costs**	$2,657,967
**Federal F&A Costs**	$1,271,452
**Approved Budget**	$3,929,419
**Total Amount of Federal Funds Obligated (Federal Share)**	$3,929,419
**TOTAL FEDERAL AWARD AMOUNT**	$3,929,419

**AMOUNT OF THIS ACTION (FEDERAL SHARE)**	$3,929,419

SUMMARY TOTALS FOR ALL YEARS		
YR	THIS AWARD	CUMULATIVE TOTALS
1	$3,929,419	$3,929,419

Fiscal Information:
CFDA Name: Nursing Research
CFDA Number: 93.361
EIN: 1223849199A1
Document Number: UNR019196A
PMS Account Type: P (Subaccount)
Fiscal Year: 2020

IC	CAN	2020
NS	8042245	$3,929,419

NIH Administrative Data:
PCC: BSMLT / **OC**: 41026 / **Released**: ALBERTINIB 08/07/2020
Award Processed: 08/14/2020 12:02:27 AM

SECTION II – PAYMENT/HOTLINE INFORMATION – 1UG3NR019196-01

For payment and HHS Office of Inspector General Hotline information, see the NIH Home Page at http://grants.nih.gov/grants/policy/awardconditions.htm

SECTION III – TERMS AND CONDITIONS – 1UG3NR019196-01

This award is based on the application submitted to, and as approved by, NIH on the above-titled project and is subject to the terms and conditions incorporated either directly or by reference in the following:

a. The grant program legislation and program regulation cited in this Notice of Award.
b. Conditions on activities and expenditure of funds in other statutory requirements, such as those included in appropriations acts.
c. 45 CFR Part 75.
d. National Policy Requirements and all other requirements described in the NIH Grants Policy Statement, including addenda in effect as of the beginning date of the budget period.
e. Federal Award Performance Goals: As required by the periodic report in the RPPR or in the final progress report when applicable.

f. This award notice, INCLUDING THE TERMS AND CONDITIONS CITED BELOW.

(See NIH Home Page at http://grants.nih.gov/grants/policy/awardconditions.htm for certain references cited above.)

Research and Development (R&D): All awards issued by the National Institutes of Health (NIH) meet the definition of "Research and Development" at 45 CFR Part§ 75.2**.** As such, auditees should identify NIH awards as part of the R&D cluster on the Schedule of Expenditures of Federal Awards (SEFA). The auditor should test NIH awards for compliance as instructed in Part V, Clusters of Programs. NIH recognizes that some awards may have another classification for purposes of indirect costs. The auditor is not required to report the disconnect (i.e., the award is classified as R&D for Federal Audit Requirement purposes but non-research for indirect cost rate purposes), unless the auditee is charging indirect costs at a rate other than the rate(s) specified in the award document(s).

Carry over of an unobligated balance into the next budget period requires Grants Management Officer prior approval.

This grant is excluded from Streamlined Noncompeting Award Procedures (SNAP).

MULTI-YEAR FUNDED AWARD: This is a multi-year funded award. A progress report is due annually on or before the anniversary of the budget/project period start date of the award, in accord with the instructions posted at: http://grants.nih.gov/grants/policy/myf.htm.

This award is subject to the requirements of 2 CFR Part 25 for institutions to receive a Dun & Bradstreet Universal Numbering System (DUNS) number and maintain an active registration in the System for Award Management (SAM). Should a consortium/subaward be issued under this award, a DUNS requirement must be included. See http://grants.nih.gov/grants/policy/awardconditions.htm for the full NIH award term implementing this requirement and other additional information.

This award has been assigned the Federal Award Identification Number (FAIN) UG3NR019196. Recipients must document the assigned FAIN on each consortium/subaward issued under this award.

Based on the project period start date of this project, this award is likely subject to the Transparency Act subaward and executive compensation reporting requirement of 2 CFR Part 170. There are conditions that may exclude this award; see http://grants.nih.gov/grants/policy/awardconditions.htm for additional award applicability information.

In accordance with P.L. 110-161, compliance with the NIH Public Access Policy is now mandatory. For more information, see NOT-OD-08-033 and the Public Access website: http://publicaccess.nih.gov/.

This award provides support for one or more clinical trials. By law (Title VIII, Section 801 of Public Law 110-85), the "responsible party" must register "applicable clinical trials" on the ClinicalTrials.gov Protocol Registration System Information Website. NIH encourages registration of all trials whether required under the law or not. For more information, see http://grants.nih.gov/ClinicalTrials_fdaaa/
This award represents the final year of the competitive segment for this grant. See the NIH Grants Policy Statement Section 8.6 Closeout for complete closeout requirements at: http://grants.nih.gov/grants/policy/policy.htm#gps.

A final expenditure Federal Financial Report (FFR) (SF 425) must be submitted through the eRA Commons (Commons) within 120 days of the period of performance end date; see the NIH Grants Policy Statement Section 8.6.1 Financial Reports, http://grants.nih.gov/grants/policy/policy.htm#gps, for additional information on this submission requirement. The final FFR must indicate the exact balance of unobligated funds and may not reflect any unliquidated obligations. There must be no discrepancies between the final FFR expenditure data and the Payment Management System's (PMS) quarterly cash transaction data.

A final quarterly federal cash transaction report is not required for awards in PMS B subaccounts (i.e., awards to foreign entities and to Federal agencies). NIH will close the awards using the last recorded cash drawdown level in PMS for awards that do not require a final FFR on expenditures or quarterly federal cash transaction reporting. It is important to note that for financial closeout, if a grantee fails to submit a required final expenditure FFR, NIH will close the grant using the last recorded cash drawdown level. If the grantee submits a final expenditure FFR but does not reconcile any discrepancies between expenditures reported on the final expenditure FFR and the last cash report to PMS, NIH will close the award at the lower amount. This could be considered a debt or result in disallowed costs.

A Final Invention Statement and Certification form (HHS 568), (not applicable to training, construction, conference or cancer education grants) must be submitted within 120 days of the expiration date. The HHS 568 form may be downloaded at: http://grants.nih.gov/grants/forms.htm. This paragraph does not apply to Training grants, Fellowships, and certain other programs—i.e., activity codes C06, D42, D43, D71, DP7, G07, G08, G11, K12, K16, K30, P09, P40, P41, P51, R13, R25, R28, R30, R90, RL5, RL9, S10, S14, S15, U13, U14, U41, U42, U45, UC6, UC7, UR2, X01, X02.

Unless an application for competitive renewal is submitted, a Final Research Performance Progress Report (Final RPPR) must also be submitted within 120 days of the period of performance end date. If a competitive renewal application is submitted prior to that date, then an Interim RPPR must be submitted by that date as well. Instructions for preparing an Interim or Final RPPR are at: https://grants.nih.gov/grants/rppr/rppr_instruction_guide.pdf. Any other specific requirements set forth in the terms and conditions of the award must also be addressed in the Interim or Final RPPR. *Note that data reported within Section I of the Interim and Final RPPR forms will be made public and should be written for a lay person audience*.

NIH strongly encourages electronic submission of the final invention statement through the Closeout feature in the Commons, but will accept an email or hard copy submission as indicated below.

Email: The final invention statement may be e-mailed as PDF attachments to: NIHCloseoutCenter@mail.nih.gov.

Hard copy: Paper submissions of the final invention statement may be faxed to the NIH Division of Central Grants Processing, Grants Closeout Center, at 301-480-2304, or mailed to:

National Institutes of Health
Office of Extramural Research
Division of Central Grants Processing
Grants Closeout Center
6705 Rockledge Drive
Suite 5016, MSC 7986
Bethesda, MD 20892-7986 (for regular or U.S. Postal Service Express mail)
Bethesda, MD 20817 (for other courier/express deliveries only)

NOTE: If this is the final year of a competitive segment due to the transfer of the grant to another institution, then a Final RPPR is not required. However, a final expenditure FFR is required and should be submitted electronically as noted above. If not already submitted, the Final Invention Statement is required and should be sent directly to the assigned Grants Management Specialist.

This award is funded by the following list of institutes. Any papers published under the auspices of this award must cite the funding support of all institutes.

National Institute Of Neurological Disorders And Stroke (NINDS)

In accordance with the regulatory requirements provided at 45 CFR 75.113 and Appendix XII to 45 CFR Part 75, recipients that have currently active Federal grants, cooperative agreements, and procurement contracts with cumulative total value greater than $10,000,000 must report and maintain information in the System for Award Management (SAM) about civil, criminal, and administrative proceedings in connection with the award or performance of a Federal award that reached final disposition within the most recent five-year period. The recipient must also make semiannual disclosures regarding such proceedings. Proceedings information will be made

publicly available in the designated integrity and performance system (currently the Federal Awardee Performance and Integrity Information System (FAPIIS)). Full reporting requirements and procedures are found in Appendix XII to 45 CFR Part 75. This term does not apply to NIH fellowships.
Treatment of Program Income:
Additional Costs

SECTION IV – NR Special Terms and Conditions – 1UG3NR019196-01

Clinical Trial Indicator: Yes
This award supports one or more NIH-defined Clinical Trials. See the NIH Grants Policy Statement Section 1.2 for NIH definition of Clinical Trial.

COOPERATIVE AGREEMENT
This award is issued as a cooperative agreement, a financial assistance mechanism in which substantial NIH scientific and/or programmatic involvement is anticipated in the performance of the activity. This award is subject to the terms and conditions of award as set forth in the SPECIAL REQUIREMENTS section of RFA-**NS-19-021,** "HEAL Initiative: Pain Management Effectiveness Research Network: Clinical Trial Planning and Implementation Cooperative Agreement (UG3/UH3 Clinical Trial Required) ", which are hereby incorporated by reference as special terms and conditions of award.

Copies of this RFA are available at http://grants.nih.gov/grants/guide/index.html or obtained from the Grants Management Contact referenced in the award.

These special Terms and Conditions of Award are in addition to, and not in lieu of, otherwise applicable OMB administrative guidelines, Federal Regulations, including DHHS Grant Administration Regulations at 42 CFR Part 52, 45 CFR Parts 74 and 92, and other DHHS, and the NIH Grants Policy Statement.

Project Scientist Contact Information:
Project Scientist: Dr. Karen Kehl
Email: karen.kehl@nih.gov

RESTRICTION:
Future NINR support for this project is contingent upon programmatic assessment of satisfactory scientific progress and successful completion of these mutually agreed upon milestones.
Failure to achieve minimally acceptable milestone recruitment levels may result in the withholding of future support and/or negotiation of an orderly close-out of this study.
Funds in the amount of $2,844,993 total costs are restricted from use for any purpose pending the written prior approval of NINR. NINR will consider the release of each year's funding based on acceptance of the annual progress report and completion of the scheduled milestones.
Yr 2: $1,057,195 total costs
Yr 3: $978,548 total costs
Yr 4: $809,250 total costs

REQUIREMENT: Participation in Annual Investigator Meetings
The NIH HEAL Initiative will require a high level of coordination and sharing between investigators. It is expected that NIH HEAL Initiative awardees will cooperate and coordinate their activities after awards are made by participating in Program Director/Principal Investigator (PD/PI) meetings, as well as in other activities.
Release of funding for future years is contingent upon the timely and satisfactory completion of the Milestones submitted by Heather Morr, Business Official, on 8.5.20, which are incorporated by reference. The grantee must submit an annual progress report that details progress towards the milestones. This report is due annually by August 31, and should be submitted via email to the grants management specialist and program official listed on the award.

In additon, and as described in the Milestones dated 8-5-20, the grantee must submit to the NINR a copy of the Protocol in advance of the recruitment of the first subject for review and

approval. Information about this Protocol should be submitted by March 1, 2021 to the Grants Management Specialist and Program Official listed on the Notice of Grant award. If this Protocol is developed prior to March 1st, it may be submitted earlier for the NINR review and approval.

REQUIREMENT: This award reflects NINR's acceptance of the revised Aims and MYF budgets submitted 4.20.20; no significant changes may be implemented without the written prior approval of the National Institute of Nursing Research.

REQUIREMENT: The awardee is required to follow the data sharing plan dated 6.11.20 and may not implement any changes in the plan without the written prior approval of the National Institute of Nursing Research.

HEAL REQUIRMENTS

This application is awarded as part of the NIH's Helping to End Addiction Long-term (HEAL) initiative, https://www.nih.gov/research-training/medical-research-initiatives/heal-initiative.

Common Data Elements

NIH guidance for collection of common data elements and data harmonization across HEAL and HEAL related clinical pain studies is applicable to this award.

Data Sharing Language

HEAL Central Data Sharing Platform Requirements

The award recipient and its collaborators must comply with all NIH HEAL Initiative Data Sharing policies (https://heal.nih.gov/about/public-access-data) established during the project period. This includes compliance with the NIH HEAL Initiative central data platform requirements and timelines developed through the HEAL consortium. It is expected that all data collected by award recipients and their collaborators, as part of the NIH HEAL Initiative, will be shared with the NIH HEAL Initiative central data platform. All data collected as part of the NIH HEAL Initiative are so collected under a Certificate of Confidentiality and entitled to the protections thereof. Institutions who receive Data and/or Materials from this award for performance of activities under this award are required to use the Data and/or Materials only as outlined by the NIH HEAL Initiative, in a manner that is consistent with applicable state and federal laws and regulations, including any informed consent requirements and the terms of the institution's NIH funding, including NOT-OD-17-109 (https://grants.nih.gov/grants/guide/notice-files/NOT-OD-17-109.html) and 42 U.S.C. 241(d). Failure to adhere to this criterion may result in enforcement actions.

Declaration of Exceptional Circumstances (DECs)

This award is funded through the NIH HEAL Initiative (https://www.nih.gov/researchtraining/medical-research-initiatives/heal-initiative). The requirements here include but not limited to, reporting requirements and data sharing are incorporated due to the need to respond to the national opioid public health crisis. As part of the response to this crisis, the NIH intends to maximize the availability of publications and the sharing of underlying data for NIH HEAL Initiative
supported research projects. Award recipients are expected to cooperate and comply with all NIH data sharing including the aforementioned central data sharing platform requirements developed for this public health emergency during the project period

REQUIREMENT: The awardee is required to follow the data and safety monitoring plan dated 4.20.20 and may not implement any changes in the plan without the written prior approval of the National Institute of Nursing Research.

REQUIREMENT:

The clinical trial(s) supported by this award is subject to the plan submitted 4.20.20 and the NIH policy on Dissemination of NIH-Funded Clinical Trial Information. The plan states that the clinical trial(s) funded by this award will be registered in ClinicalTrials.gov not later than 21 calendar days after enrollment of the first participant and primary summary results reported in ClinicalTrials.gov, not later than one year after the completion date. The reporting of summary results is required by this term of award even if the primary completion date occurs after the period of performance.

This award is subject to additional certification requirements with each submission of the Annual, Interim, and Final Research Performance Progress Report (RPPR). The recipient must agree to the following annual certification when submitting each RPPR. By submitting the RPPR, the AOR signifies compliance, as follows:

In submitting this RPPR, the SO (or PD/PI with delegated authority), certifies to the best of his/her knowledge that, for all clinical trials funded under this NIH award, the recipient and all investigators conducting NIH-funded clinical trials are in compliance with the recipient's plan addressing compliance with the NIH Policy on Dissemination of NIH-Funded Clinical Trial Information. Any clinical trial funded in whole or in part under this award has been registered in ClinicalTrials.gov or will be registered not later than 21 calendar days after enrollment of the first participant. Summary results have been submitted to ClinicalTrials.gov or will be submitted not later than one year after the completion date, even if the completion date occurs after the period of performance.

REQUIREMENT: COMMITMENT OVERLAP
A review of Other Support information provided in the application for this project indicates that with the award of this project, the effort commitment of Dr. Frances Keefe may exceed twelve person months of effort. If applicable, the grantee is responsible both for eliminating this over-commitment (and any other over-commitment of effort) and for obtaining appropriate prior approval(s) in accordance with NIH and institutional policy requirements.

INFORMATION: CONSORTIUM/CONTRACTUAL COSTS
This award includes Year 1 funds for consortium activity with Northwestern University awarded in the amount of $69,127 total costs ($43,751 direct costs and $25,376 F&A costs). Each consortium is to be established and administered in accordance with the NIH Grants Policy Statement (revised 10/2019). No foreign performance site may be added to this project without the written prior approval of the National Institute of Nursing Research

INFORMATION: CONSORTIUM/CONTRACTUAL COSTS
This award includes funds for consortium activity with Duke University awarded in the amount of $39,421 total costs ($24,485 direct costs and $14,936 F&A costs). Each consortium is to be established and administered in accordance with the NIH Grants Policy Statement (revised 10/2019). No foreign performance site may be added to this project without the written prior approval of the National Institute of Nursing Research.

STAFF CONTACTS

The Grants Management Specialist is responsible for the negotiation, award and administration of this project and for interpretation of Grants Administration policies and provisions. The Program Official is responsible for the scientific, programmatic and technical aspects of this project. These individuals work together in overall project administration. Prior approval requests (signed by an Authorized Organizational Representative) should be submitted in writing to the Grants Management Specialist. Requests may be made via e-mail.

Grants Management Specialist: Kelli Oster
Email: osterk@mail.nih.gov **Phone**: 301.594.2177

Program Official: Lois Tully
Email: tullyla@mail.nih.gov **Phone**: 301-594-5968

SPREADSHEET SUMMARY
GRANT NUMBER: 1UG3NR019196-01

INSTITUTION: WAKE FOREST UNIVERSITY HEALTH SCIENCES

Budget	Year 1
Salaries and Wages	$1,812,327
Fringe Benefits	$329,170
Personnel Costs (Subtotal)	$2,141,497
Consultant Services	$15,300
Materials & Supplies	$10,085
Travel	$10,000
Other	$84,850
Subawards/Consortium/Contractual Costs	$396,235

TOTAL FEDERAL DC	$2,657,967
TOTAL FEDERAL F&A	$1,271,452
TOTAL COST	$3,929,419

Facilities and Administrative Costs	Year 1
F&A Cost Rate 1	55%
F&A Cost Base 1	$661,857
F&A Costs 1	$364,021
F&A Cost Rate 2	55%
F&A Cost Base 2	$620,161
F&A Costs 2	$341,089
F&A Cost Rate 3	55%
F&A Cost Base 3	$569,428
F&A Costs 3	$313,185
F&A Cost Rate 4	55%
F&A Cost Base 4	$460,286
F&A Costs 4	$253,157
